# Step-by-step approach: Stereotaxic surgery for *in vivo* extracellular field potential recording at the rat Schaffer collateral-CA1 synapse using the eLab system

**DOI:** 10.1016/j.mex.2023.102544

**Published:** 2023-12-29

**Authors:** Mansour Azimzadeh, Mohd Amirul Najwa Mohd Azmi, Parham Reisi, Pike-See Cheah, King-Hwa Ling

**Affiliations:** a Department of Biomedical Sciences Department of Human Anatomy, Faculty of Medicine and Health Sciences, Universiti Putra Malaysia, UPM Serdang, Selangor 43400, Malaysia; b Deputy Dean's Office (Research and Internationalization), Faculty of Medicine and Health Sciences, Universiti Putra Malaysia, UPM Serdang, Selangor 43400, Malaysia; c Department of Physiology, School of Medicine, Isfahan University of Medical Sciences, Isfahan, Iran; d Department of Human Anatomy, Faculty of Medicine and Health Sciences, Universiti Putra Malaysia, UPM Serdang, Selangor 43400, Malaysia; e Malaysian Research Institute on Ageing (MyAgeing™), Universiti Putra Malaysia, UPM Serdang, Selangor 43400, Malaysia

**Keywords:** CA1, Hippocampus, Local field potential measurement, Stereotaxic techniques, Schaffer collaterals, *In vivo* hippocampal extracellular local field potentials recording

## Abstract

*In vivo* extracellular field potential recording is a commonly used technique in modern neuroscience research. The success of long-term electrophysiological recordings often depends on the quality of the implantation surgery. However, there is limited use of visually guided stereotaxic neurosurgery and the application of the eLab/ePulse electrophysiology system in rodent models. This study presents a practical and functional manual guide for surgical electrode implantation in rodent models using the eLab/ePulse electrophysiology system for recording and stimulation purposes to assess neuronal functionality and synaptic plasticity. The evaluation parameters included the input/output function (IO), paired-pulse facilitation or depression (PPF/PPD), long-term potentiation (LTP), and long-term depression (LTD).

• Provides a detailed picture-guided procedure for conducting *in vivo* stereotaxic neurosurgery.
• Specifically covers the insertion of hippocampal electrodes and the recording of evoked extracellular field potentials.

## Graphical abstract

**Figure:**
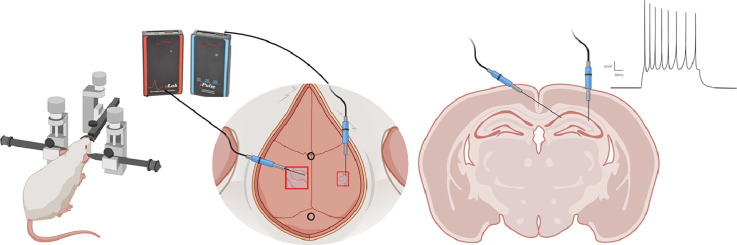

Specifications Table

**Table:** 

Subject area:	Neuroscience
More specific subject area:	*In vivo* electrophysiology
Name of your method:	*In vivo* hippocampal extracellular local field potentials recording
Name and reference of original method:	Stereotaxic surgery and hippocampal extracellular local field potential recording. doi: 10.1016/j.eplepsyres.2022.107064
Resource availability:	Surgical tools, stereotaxic device, animal, anesthetic and analgesic drugs, stainless steel electrode, electrophysiology stimulatory and recording device, laptop.

## Method details

### Electrophysiology

**Fig. 1:**
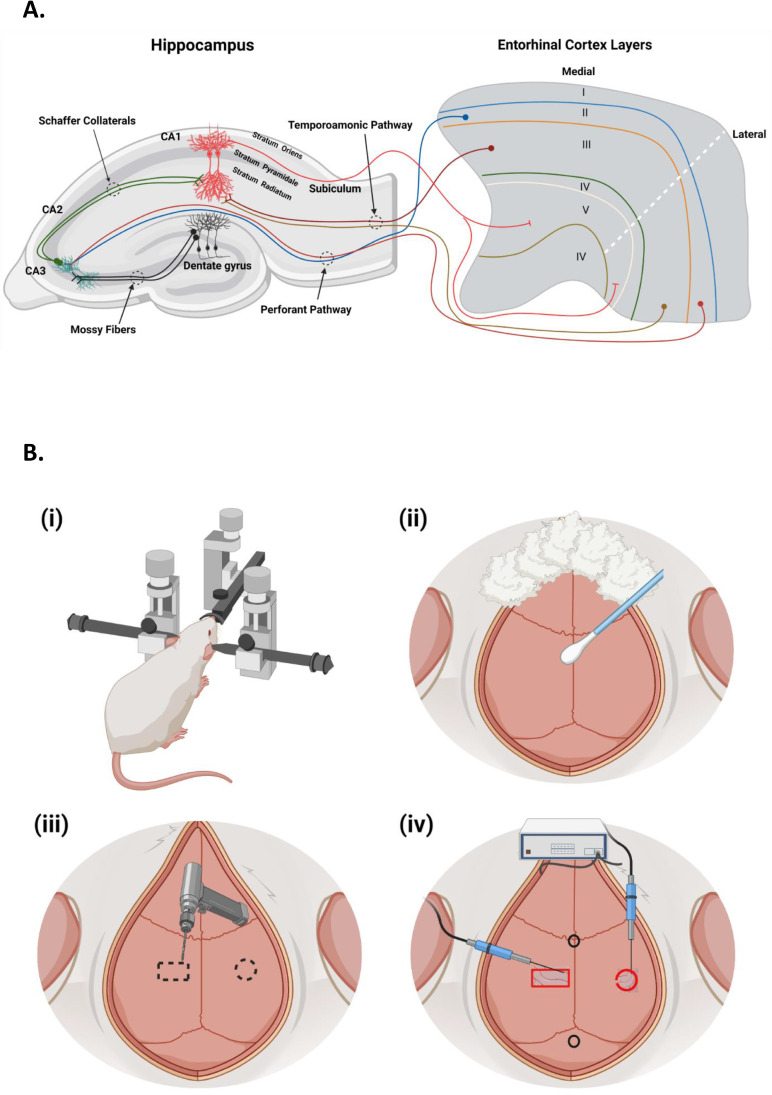
**(A)** Illustrates the anatomy of the hippocampus and entorhinal cortex pathways. The axons of pyramidal cells from the entorhinal cortex's second layer (II) enter the hippocampus and synapses with the granule cells of the DG and the pyramidal cells of the CA3 region, forming the Perforant pathway. The axons of the granule cells of the DG (Mossy Fiber) then synapse with the pyramidal cells of the CA3 region. The axons from CA3 pyramidal cells (Schaffer collaterals) enter the CA1, synapsing with the proximal dendrites of the CA1 area. The CA1 axons then project back to the deeper layer of the entorhinal cortex (layer V), creating an anatomical and functional loop. Also, the axons from layer III of the entorhinal cortex (Temporoamonic pathway) enter the CA1 and synapse with the distal dendrites. **(B)** The figure also includes a schematic view of (i) an animal placed onto a stereotaxic device, (ii) the exposed skull, determination of the bregma and lambda, finding the coordinate of Schaffer collaterals and CA1, (iii) drilling the marked location, and (iv) insertion of both stimulatory and recording electrodes. This figure was created with BioRender.com.

Electrophysiology is a field of study involving the electrical properties of biological cells and tissues, from ion channels to whole organs like the brain and heart. Field potential recording measures potential differences near cells using a microelectrode [1,2]. The hippocampus in the temporal lobe is crucial for learning and memory [3,4] (Fig. 1A). Long-term potentiation (LTP) strengthens synapses and is essential for synaptic plasticity, learning, and memory (5).

**Fig. 2:**
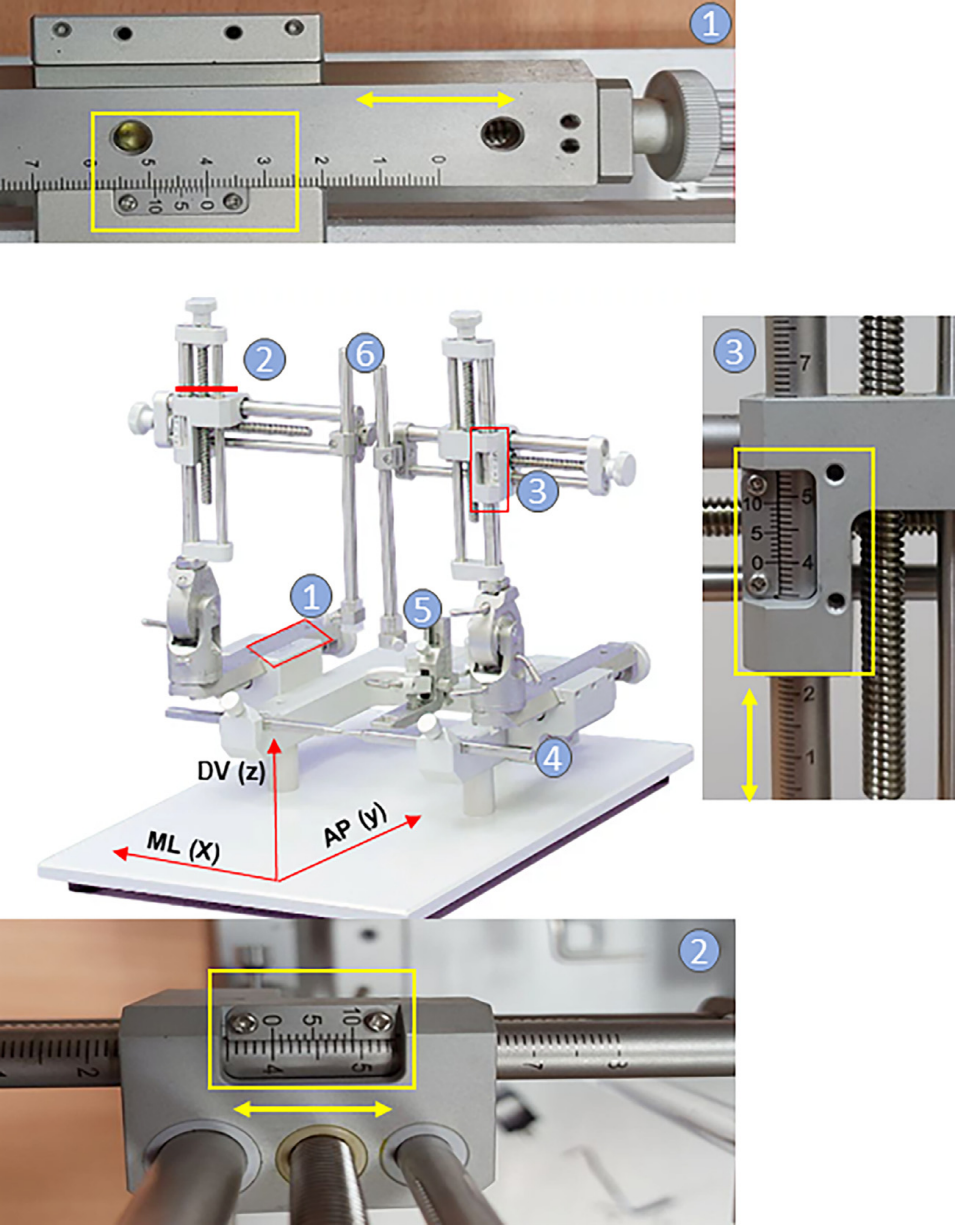
The two-arm stereotaxic device is equipped with three micromanipulators that enable movement in three dimensional spaces along the X (medio-lateral/ML), Y (antero-posterior/AP), and Z (dorso-ventral/DV) axes. The device includes microdrive screw adjustments for the AP, ML, and DV axes ([1], [2], [3]), also ear bars (4), an incisor bar (5), and two holders for left and right device arms (6).

Careful planning is required when implanting electrodes into specific areas of the brain. A stereotaxic device with three micromanipulators is used to move the electrodes in three-dimensional space (X, Y, and Z axes) (Fig. 2). The brain atlas is used as a reference for orientation and assigning a location to experimental data. This precision is crucial for successfully implanting the electrodes and conducting effective electrophysiological experiments (Fig. 1B).

ELab (Science Beam, Parto Danesh Co., Tehran, Iran) is a comprehensive electrophysiology workstation that serves as a data acquisition system, allowing for simultaneous recording of various extracellular potentials. It can also modulate signals through external digital events and includes a compatible pulse generator for customized electrical stimulation protocols (https://sciencebeam.com/elab/) [[7], [8], [9], [10], [11]].

We present a comprehensive guideline for measuring electrophysiological signals from the rat hippocampus using the eLab/ePulse system. Our guide includes determining stereotaxic coordinates, performing neurosurgery, and inserting electrodes. We also provide step-by-step instructions for executing specific protocols and analyzing data. This approach simplifies in vivo electrophysiology experiments and eliminates the need for extensive technical expertise.

### Animals

In this study, male adult Wistar rats weighing ∼250 g was utilized. All usage of animals in this study was approved by the Universiti Putra Malaysia Institutional Animal Care and Use Committee (IACUC) (Approval reference: UPM/IACUC/AUP-R033/2021).

### Pre-recording procedures

#### Surgeries

The animal was injected intraperitoneally (i.p.) with urethane (Sigma, CAS Number: 51-79-6) at a dosage of 1.6 g/kg for rats. If necessary, one-tenth of the initial dose was administered for maintenance. The stereotaxic frame and necessary materials were set up and sterilized. The top of the animal's head was shaved using an electric razor, and then scrubbed with isopropyl alcohol followed by povidone/iodine. Ophthalmic ointment and an eye cover were applied to prevent dry eyes.

**Fig. 3:**
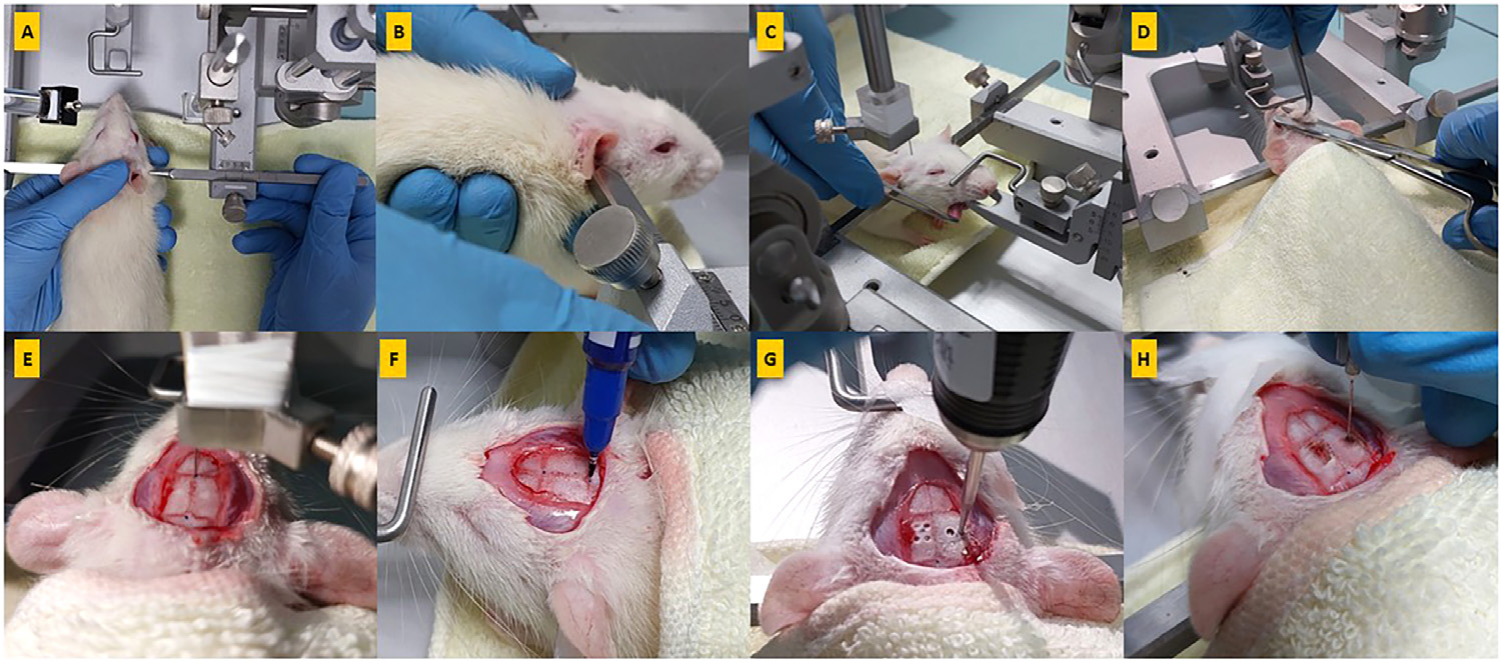
A picture-guided neurosurgery was performed prior to electrode insertion. **(A)** The rat was mounted onto a two-arm stereotaxic device, with the left side ear bar in place, while the left hand supports the animal. **(B)** The right ear bar was then loosened and placed into the auditory canal. **(C)** The rat's tongue was retracted to the side using forceps. **(D)** The scalp was excised using fine scissors; **(E, F)** the bregma (Br) and lambda (La) were identified and temporarily marked using a pointer (gauge 27); **(G)** a dental micromotor hand drill was used to perform craniotomy and make four pilot holes at the corners of the marked locations (right side for Schaffer collaterals, and left side for CA1) and; **(H)** the dura mater was pierced using a needle (gauge 27).

After evaluation the depth of anesthesia using tail and toe pinch withdrawal reflexes, the animal was mounted onto the stereotaxic device by inserting the ear bars into the auditory canal. To do this, the left ear bar was held in place while the right ear bar was inserted into the auditory canal (Fig. 3A, B). The correct position of the ear bars was confirmed by the corneal blinking reflex. The tongue of the rat was then retracted to the side using forceps, and the incisor bar was placed between the upper and lower jaws (Fig. 3C). The scalp was excised using fine scissors (Fig. 3D). The periosteum connective tissue was gently removed using a dental scraper. The exposed skull surface was dried to enhance the visibility of the bregma and cranial sutures.

#### Finding coordination of the desired location within the brain

A guide cannula (gauge 27 or 28) was used to identify and mark two reference points: bregma is the intersections of the sagittal suture with the coronal suture, while lambda is the intersection of the sagittal suture with the lambdoidal suture (Fig. 3E, F). The coordinates of the anterior-posterior (AP) and mediolateral (ML) positions of both points were recorded. To calculate the AP difference between bregma and lambda (AP_Br_ - AP_La_), the AP coordinate of bregma was subtracted from the AP coordinate of lambda (AP_Br_ - AP_La_).

If the obtained data is 9.1 ± 0.3 (based on a male Wistar rat weighing 290 g), no correction coefficient (CC) is needed. However, if the data differs from this range a CC should be applied to determine the desired location coordinates.

### Correction coefficient (CC) for Schaffer collaterals and CA1

The desired locations for both stimulation and recording were the right side of the hippocampal Schaffer collaterals and the CA1 respectively. According to the Paxinos atlas, the coordinates for the right Schaffer collaterals are AP= -4.2, ML= +3.8, DV= 2.7 – 3.8 (from the dura) and for the right CA1, the coordinates are AP= -3.4, ML= +1.5, DV= 4.4 – 5.1 (from the dura). If the AP_Br_ is 47.5 and the AP_La_ is 39.2, the difference between AP_Br_ and AP_La_ is 8.3, which is less than 9.1 ± 0.3. Therefore, it is necessary to apply the CC for the AP of Schaffer collaterals and CA1 coordinates separately.

If:

AP_Br_= 47.5 & AP_La_= 39.2, therefore, AP_Br_ - AP_La_= 8.3 < 9.1 ± 0.3.

It is necessary to apply the CC for both the AP of both Schaffer collaterals and CA1 coordinates separately.

**Table:** 

Schaffer collaterals	CA1
$\frac{9.1}{8.3}=\;\frac{-4.2}{x}\;\to \;x=-3.8$	$\frac{9.1}{8.3}=\;\frac{-3.4}{x}\;\to \;x=-3.1$

The value of “9.1 ± 0.3” represents the distance between AP_Br_ - AP_La_ in a male Wistar rat weighing 290 g, according to the Paxinos atlas_._ The value of "8.3̎ is the distance between AP_B_ - AP_L_ obtained from a specific rat. The values of “-4.2” and “-3.4” indicate the AP coordination of the Schaffer collaterals and CA1 respectively, based on the Paxinos atlas [12].

### Electrode insertion procedures for Schaffer collaterals / CA1

**Fig. 4:**
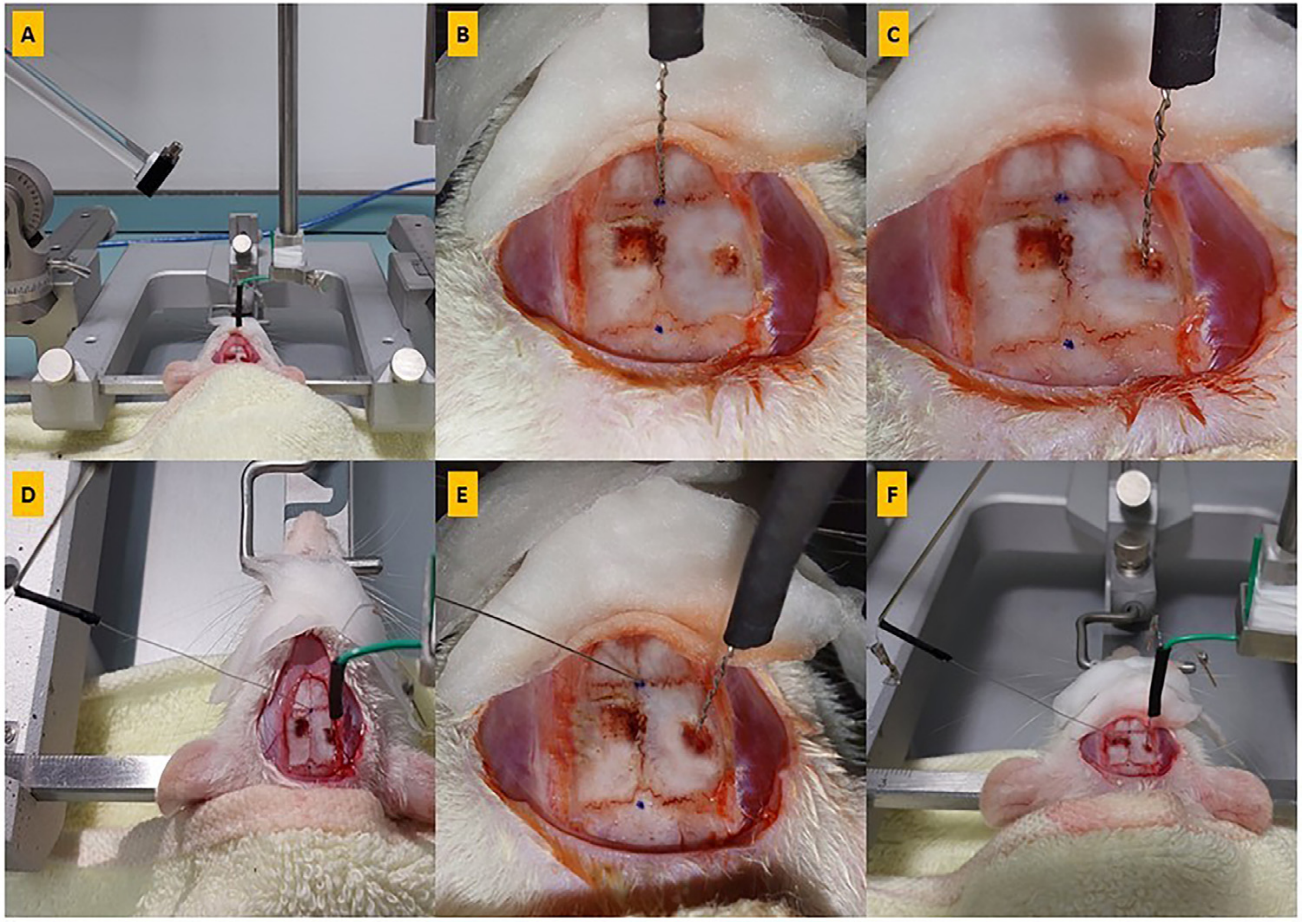
Inserting stimulation and recording electrodes. **(A)** Replace the guide cannula with the bipolar stimulation electrode on the right arm of the stereotaxic frame; **(B)** Place the tip of the stimulation electrode on the bregma; **(C)** Insert the stimulation electrode after calculating the coordinates; **(D)** Place the recording electrode on the left arm of the device (angled at 52.5 degrees); **(E)** Place the tip of the recording electrode on the bregma; **(F)** Insert the recording electrode after calculating the coordinates.

This procedure consists of two steps: a) determining the Schaffer collaterals coordinates and b) determining the CA1 coordinates. First, the bregma was located and the AP and ML coordinates were recorded using a guide cannula., The coordination of Schaffer collaterals with the original bregma location was determined using the AP (X) and ML (Y) axes of the stereotaxic device. Then, the guide cannula was lowered along the DV axis until it touched the skull and marking drilling area. The coordination of CA1 with the original bregma location determined and marked. four pilot holes were made at the corners of the marked locations using a dental micromotor hand drill (Fig. 3G). The craniotomy should be limited to a smaller area (≈ 2-3 mm). The perimeter of the craniotomy was drilled to remove the bone mass in the center. During surgery, it is important to avoid the superior sagittal sinus, which may be located within 0.5 mm of the midline longitudinal suture and is a major blood vessel. Saline or artificial CSF was used to keep the exposed dura hydrated. The guide cannula was then removed and replaced with the bipolar stimulation electrode on the right arm of the stereotaxic frame (Fig. 4A). In electrophysiology experiments, two main types of electrodes can be used: glass micropipettes and metal electrodes (steel, tungsten, or platinum). Typically, only metal electrodes are used for stimulation, while both metal and glass electrodes can be used for recording. In the present study, Teflon-coated stainless-steel electrodes with a diameter of 0.125 mm (Advent Co., UK) were used.

To measure the coordinates of the right hippocampal Schaffer collaterals, we first placed the tip of the electrode above the bregma (see Fig. 4B). We recorded and calculated the AP and ML data. Then, we positioned the electrode at the obtained AP and ML coordinates. Using the DV axes (Z), we lowered the electrode holder until the tip of the electrode touched the surface of the dura. We recorded and calculated the value of DV. To facilitate electrode insertion, we gently pierced the dura mater with a sterile hypodermic needle of a small gauge [[13], [14]] or a small hook [15] (see Fig. 3H). We slowly advanced the stimulation electrode into the brain at a rate of 1 mm every 10 seconds) until reaching the calculated depth (see Fig. 4C). Finally, we secured the recording electrode on the left arm of the stereotaxic frame, angulated at 52.5 degrees [16] (see Fig. 4D).

To measure the coordinates of the right hippocampal CA1, we first positioned the tip of the electrode above the bregma. We recorded and calculated the AP and ML data (Fig. 4E). Then, we adjusted the electrode to the obtained AP and ML coordinates. Using the DV axes (Z), we lowered the electrode holder until the tip of the electrode touched the surface of the dura. We recorded the value of DV. To facilitate electrode insertion, we gently pierced the dura mater with a sterile hypodermic needle of a small gauge [[13], [14]] or a small hook. We slowly advanced the electrode from the left hemisphere into the right brain hemisphere at a rate of 1 mm every 10 seconds until reaching the desired depth (Fig. 4F).

### Design protocol

A digital signal is composed of digital values that are converted from an analog signal at specific time intervals. In contrast, an analog signal is continuous in both time and amplitude. Digital signals are represented by numerical sequences, with time being indicated by the sequence's index. Time in digital signals is considered discrete or discontinuous. Digital signals can be generated artificially through algorithms or by converting analog signals to digital signals using analog-to-digital converters (ADC) [17].

In electrophysiology experiments, the signals received by electrodes are initially analog. These analog signals are then amplified by an amplifier before being sent to either an analog-to-digital converter or a computer with software. The waveform is then digitized, represented by zeros and ones, and displayed through a digital waveform interface (https://sciencebeam.com/elab/).

This article provides instructions on how to utilize the eLab stimulation and recording devices.

**Fig. 5:**
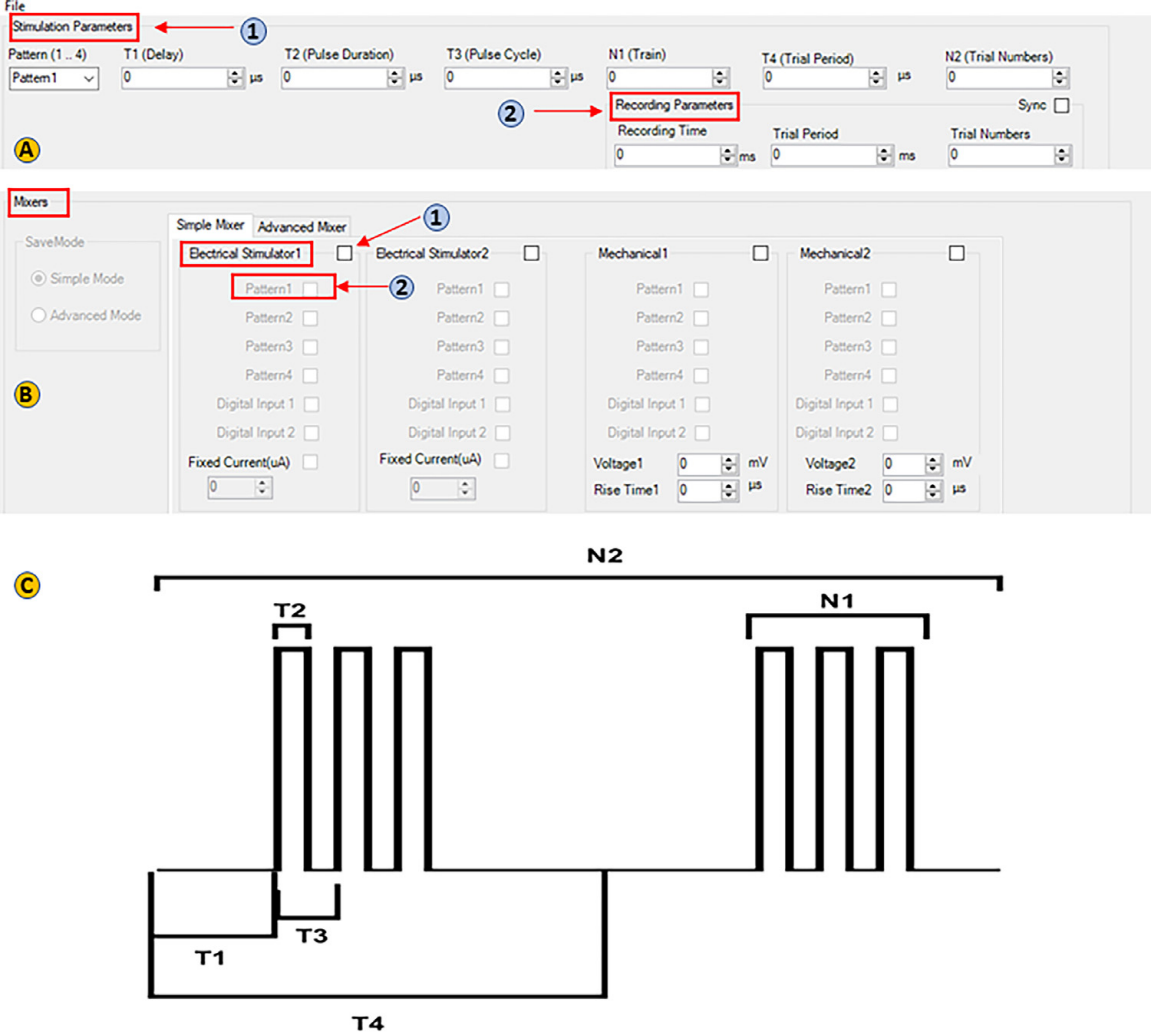
View the eLab design protocol page for the followingparameters: **(A-1)** Stimulation parameters. **(A-2)** Recording parameters. **(B)** Mixer section to activate stimulator. Refer to the text for more details. **(C)** A putative waveform with the definition of the protocol parameters.

The “Design Protocol” tab on the main page allows users to create various patterns for stimulation and recording. There are two main sections: 1) stimulation parameters and 2) recording parameters. In the “Stimulation parameters” section (Figs. 5A-1, C), users can find the following options:

• T1 (Delay) refers to the time delay between starting the recording and applying the first stimulation pulse in each train.
• T2 (Pulse Duration) is the duration of a single stimulation pulse.
• T3 (Pulse Cycle) is the duration between the start of one pulse and the start of the next pulse.
• N1 (Train) presents the number of pulses in a trial period.
• T4 (Trial Period) is the duration between the start of one trial period and the start of the next one.
• N2 (Trial Numbers) determines how many times a desired trial should be repeated.

The "Recording parameter" (Fig. 5A-2) includes the following:

• Recording time is a part of the Trial Period (T4) and allows for saving the events displayed on the monitor.
• The Trial Period and Trial Numbers are the same as described above. The recording time should not exceed the trial period (it can be equal to or shorter than the trial period).
• To ensure consistency, check the sync box to align the values of the Trial period and Trial Numbers in both Menu bars.
• In the mixer section, check the "Electrical Stimulator 1̎ box (Fig. 5B-1) to activate the stimulator. Next click the pattern 1 box (Fig. 5B-2) to integrate the stimulator with the protocol.
• It is important to note that time in the stimulator is measured in microseconds, while in the recording it is measured in milliseconds.

#### Pattern test protocol

The pattern test protocol is necessary to determine the optimal location for both the stimulation and recording electrodes, which is determined by the largest evoked field excitatory postsynaptic potential (fEPSP).

To design a pattern test protocol, the parameters for both stimulation and recording sections are as follows (Supplementary Fig. 1B):

rst T1 = 10000 µs, T2 = 150 µs, T3 =1000 µs, T4 = 10000000 µs, N1 = 1, N2 = 100; and recording time= 0 ms, trial period = 1000 ms, trial number = 100.

#### Input/output (IO) protocol

The IO curve reflects the neuronal excitability of a stimulation target curve and is influenced by neuromodulators, incoming endogenous signals, the brain state, and various illnesses [18]. Extensive research is being conducted to investigate the association between IO curve characteristics and medical disorders. For example, IO curves can serve as indicators for epilepsy [19], dementia [20], and neurodegenerative illnesses [21]. The IO curve provides a comprehensive stimulus-response pattern including elements like maximum recruitment and physiological variability from different sites and mechanisms. It ranges from weak stimuli with minimal response to high pulses where the response saturates [18]. Moreover, the IO functional curve is a characteristic of well-established synapses and helps determine the desired stimulus intensity.

The general IO protocol for both the stimulation and recording sections is as follows (Supplementary Fig. 1C):

rst T1 = 10000 µs, T2 = 150 µs, T3 = 1000 µs, T4 = 10000000 µs, N1 = 1, N2 = 10; and recording time = 500 ms, trial period = 10000 ms, trial number = 10.

#### Paired pulse protocol

The short-term forms of activity-dependent synaptic plasticity are referred to as paired-pulse facilitation (PPF) and depression (PPD) events. PPF and PPD are commonly used as a model to assess the presynaptic component of synapses, which exhibit an increase in the amplitude of the second evoked EPSPs following the first one with a short interstimulus interval [22]. This increase in the probability of neurotransmitter release is mainly attributed to residual Ca^2+^ in the nerve terminals after the initial stimulus. For designing a paired-pulse protocol, the parameters for both stimulation and recording sections are as follows (Supplementary Fig. 2C–E):

rst T1 = 10000 µs, T2 = 150 µs, T3 “X” µs, T4 = 10000000 µs, N1 = 2, N2 = 5; and recording time = 500 ms, trial period = 10000 ms, trial number = 5.

The pulse cycle, referred to as T3, is the duration between the start of pulse and the start of the next pulse. Therefore, when determining the paired pulse, it is important to consider the time between two evoked EPSPs.

The IO protocol has a fixed protocol with varying stimulus currents (ranging from 100-1000 µA), while paired-pulse protocols have a fixed stimulus current (based on the IO curve) and varying pulse cycles. For example, if the distance between two evoked EPSPs is 10 ms, the T3 value would 10000 µs. Therefore, for different intervals of evoked EPSPs (10, 20, 30, 50, 70, and 100 ms), a distinct protocol needs to be designed with different T3 values.

#### Baseline 30 and 90 minutes

A 30-minute baseline fEPSP recording is necessary to stabilize the neurons in the recording area before inducing LTP/LTD. After induction, a 90-min baseline fEPSP is required to compare the percentage changes in two main fEPSP characteristics: amplitude and slope from the initial baseline.

To design the 30-minute baseline protocol, the parameters for both the stimulation and recording sections are as follows (Supplementary Fig. 1D, E):

rst T1 = 10000 µs, T2 = 150 µs, T3 = 1000 µs, T4 = 10000000 µs, N1 = 1, N2 = 180; and recording time = 500 ms, trial period = 10000 ms, trial number = 180.

It is important to note that the 30-minute baseline protocol consists of 180 stimuli, assuming that each minute is equivalent to 60 seconds and one stimulus is delivered every 10 seconds. The parameters of the baseline 90-minute protocol are the same as the 30-minute protocol except for the N2 and trial number items which are both set to "540".

#### LTP and LTD protocols

LTP and LTD are essential for understanding synaptic plasticity, a key feature of the nervous system, that is closely associated with learning and memory [23]. LTP and LTD are two types of synaptic plasticity that occur at the same synapse in response to different patterns of NMDA receptor activation. Despite having opposing effects on synaptic excitability, both LTP and LTD contribute to the process of learning and memory. Long-term synaptic plasticity refers to a lasting change in the strength or efficacy of synaptic transmission that is dependent on activity [24]. It can enhance bidirectionally change synaptic strength through LTP or depress it through LTD [25].

To design an LTP 100 Hz protocol, we need to consider the parameters for both the stimulation and recording sections are as follows (Supplementary Fig. 2A):

rst T1 = 10000 µs, T2 = 150 µs, T3 = 10000 µs, T4 = 10000000 µs, N1 = 50, N2 = 4; and recording time = 0 ms, trial period = 10000 ms, trial number = 4.

To design an LTD 1 Hz protocol, both stimulation and recording sections are as follows (Supplementary Fig. 2B):

rst T1 = 10000 µs, T2 = 150 µs, T3 = 1000 µs, T4 = 1000000 µs, N1 = 1, N2 = 900; and recording time = 0 ms, trial period = 1000 ms, trial number = 900.

In all protocols, it is important to click on “Electrical Stimulator 1” and then select “pattern 1” from the Mixers section on the “Design protocol” page. This will activate the stimulator for designed protocol.

### Step-by-step to run the designed protocols and recording fEPSP

(1) Turn on the laptop.
(2) Connect the eLab to the laptop and plug in the ePulse using a power adaptor (output 5.0V, 1.55A) to provide a low-level DC voltage and current.
(3) Adjust the following settings in from the scope window of the eLab environment by right-clicking: V/D: 200 or 500 µV, T/D: 5ms, HPF: 1 Hz, LPF: 3 KHz, Trigger: on (Supplementary Fig. 1A).
(4) Select the pattern test from the “Experiment protocol” browser tab and save the file from “Recording file” tab.
(5) Set the stimulator current to 300 µA.
(6) Start the protocol.
(7) Slowly move the electrodes within the obtained DV range of both the stimulation and recording electrodes. A favourable movement will be carried out in search of the maximum response of evoked fEPSP.
(8) Allow for a temporary halt of at least 30 minutes to allow for the recovery of the stimulated area neurons.
(9) Choose the IO protocol from the “Experiment protocol” browser tab.
(10) Set the stimulator current to 100 µA and save the file named IO100 from the recording file tab. Repeat step 6.
(11) Repeat Step 10 for currents ranging from 200 µA up to 1000 µA (increasing intensities in increments of 100 µA) and save the corresponding files for each. It is recommended to wait 3 to 5 minutes between consecutive stimulations and protocols.
(12) Adjust the intensity of the test stimuli after recording the IO function to achieve 50% of the maximum response of the evoked fEPSP slope or amplitude for the paired-pulse, baseline responses, LTP, and LTD functions.
(13) Choose the Paired pulse 10 protocol and save it as Paired pulse 10. Repeat step 6.
(14) Follow step 12 by choosing the appropriate protocol for each paired-pulse 20, 30, 50, 70, and 100 and save the corresponding files for each.
(15) Choose the baseline 30-minute protocol and save the file with the same name. Repeat step 6.
(16) Choose the LTP 100 Hz protocol and save the file with the same name. Repeat step 6.
(17) Choose the baseline 90-minute protocol and save the file with the same name. Repeat step 6.
(18) For applying LTD, repeat step 15.
(19) Choose the LTD 1 Hz protocol and save the file with the same name. Repeat step 6.
(20) Choose the baseline 30-minute protocol and save the file with the same name. Repeat step 6.

### Histology

**Fig. 6:**
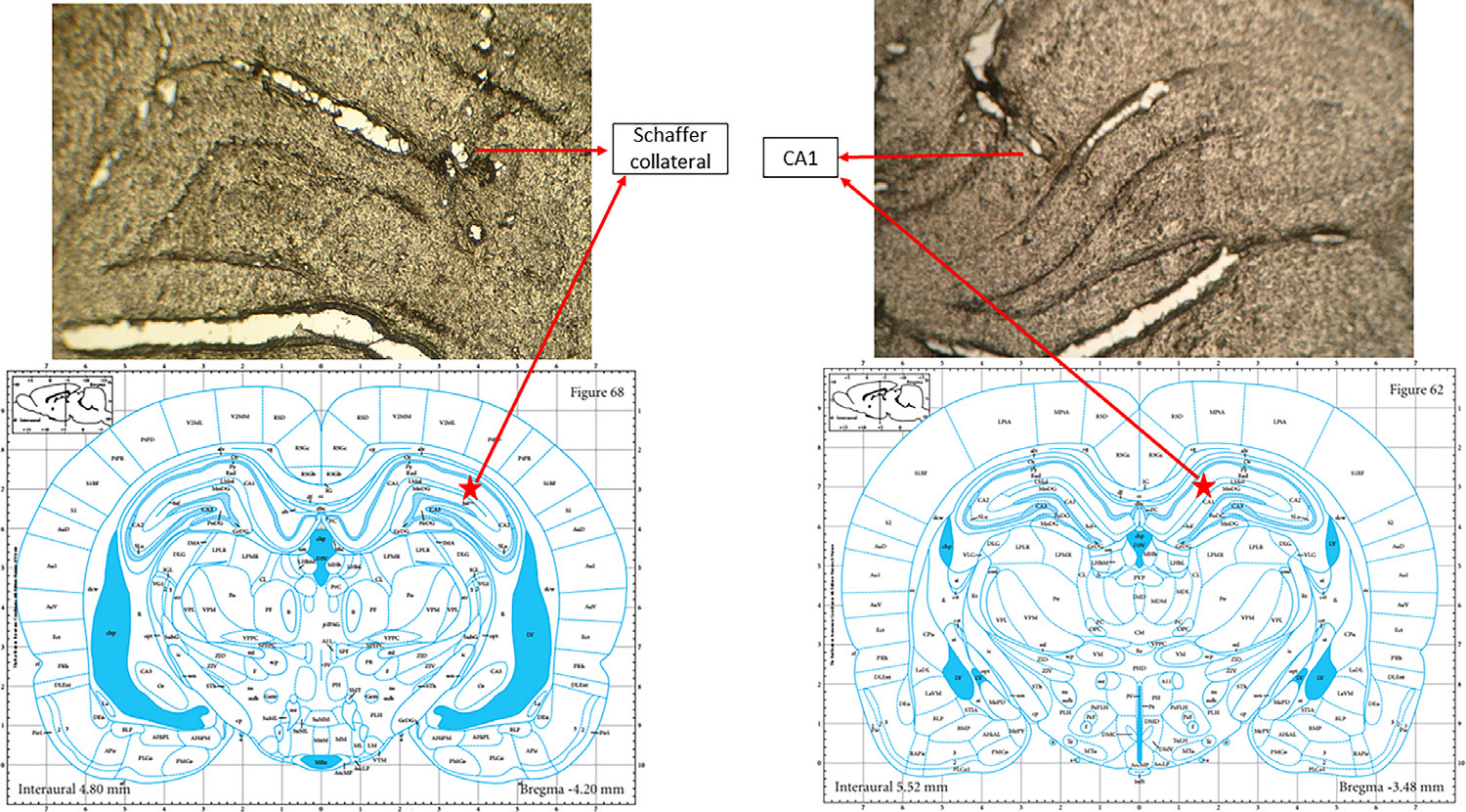
Histological image of the right hippocampus. In the top photos, the stimulatory electrode was placed on the Schaffer collaterals (*left*) and the recording electrode was placed on CA1 (*right*). In the bottom photos, red stars indicate the coordinates of Schaffer collaterals (*left*) and CA1 (*right*) on the Paxinos atlas.

The rat was perfused transcranially with saline, followed by 10% buffered formalin. The brain was then removed and fixed in 10% buffered formalin for 2-3 days. The fixed tissues were sliced into coronal sections that were 55 µm thick. This was done to histologically verify the position of the electrodes at the end of the electrophysiological investigation using a light microscopic approach (Fig. 6).

## Method validation

**Fig. 7:**
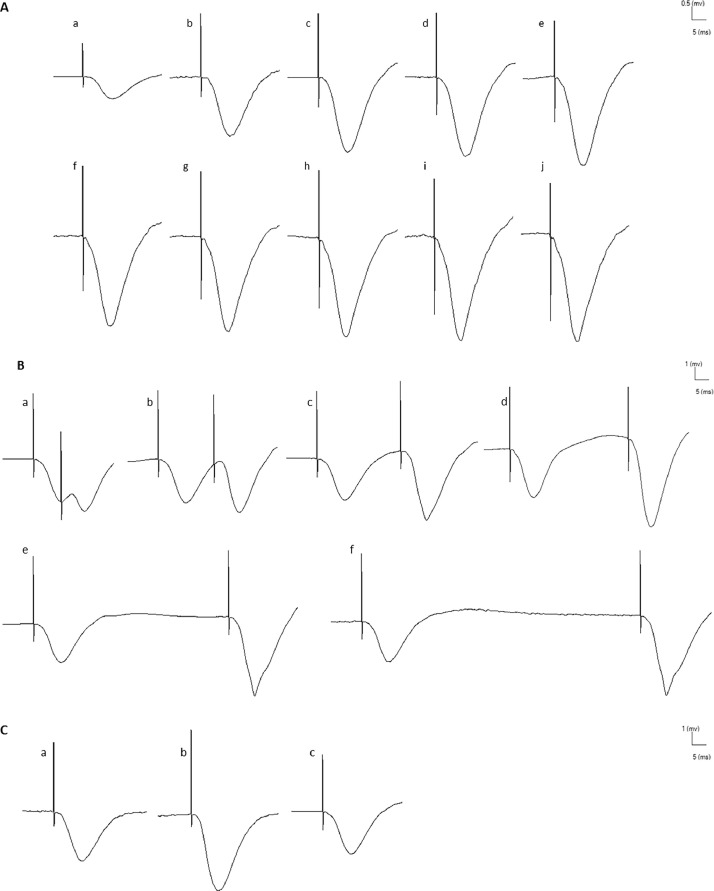
**(A)** EPSPs recorded from the IO 100-1000, with 10 different intensities labeled as a-j. **(B)** Paired pulses were recorded at intervals of 10, 20, 30, 50, 70 and 100 ms, labeled as a-f. **(C)** EPSPs were recorded before LTP / LTD induction (a), after LTP induction (b), and after LTD induction (c).

The eProbe /eTrace analysis program allows for offline primary data detection and extraction, as well as figure export. The IO function curve is obtained by measuring the averages of ten test pulses applied at each current intensity (100-1000 µA). The amplitude and slope of the EPSP increase with higher current intensities (Fig. 7A). The paired pulses curve is obtained by measuring the averages of five test pulses applied at different interstimulus intervals (10,20,30,50,70, and100) separately (Fig. 7B). The percentage change of the second pulse compared to the first pulse is then calculated. To evaluate LTP and LTD, the EPSPs are measured before and after induction (Fig. 7C). The baseline at 30 minutes is determined by averaging the evoked fEPSPs every 5 minutes, resulting in six points on the curve. The baseline at 90 minutes is determined by averaging the evoked fEPSPs every 10 minutes, resulting in nine points on the curve.

The percentage change of the fEPSP slope and amplitude at baseline 90 minutes after LTP induction is compared to the initial baseline at 30 minutes. To evaluate LTP induction, two parameters, induction and maintenance, are assessed. Usually, induction is considered to occur when the fEPSP changes by at least 20%. For LTD evaluation, the percentage change of the fEPSP slope or amplitude at baseline 30 minutes after LTD induction is compared to the baseline at 30 minutes.

Stereotaxic neurosurgery is a successful technique used on rodents for neuroscience studies. It allows for the introduction of electrodes into specific brain regions for studying neuroplasticity and brain networks in behaving animals. The main objective of this study was to optimize a surgical method using a picture-guide surgical method to improve the precision and efficiency of these complex surgeries. The aim was to minimize experimental errors, reduce the number of animals used, and maximize the quality of the experimental data. By following this high-precision picture-guide, not only can non-veterinarians or non-anatomists perform craniotomy, but neuroscientists can also benefit from the technical refinements suggested in the guide. The entire stereotaxic neurosurgery procedure using this method takes approximately 30 minutes per rat.

This study aimed to improve surgical precision and efficiency using a picture-guide surgical method, reducing experimental errors and the number of animals used while maximizing data quality. The guide allows non-experts to perform craniotomy and provides technical refinements for neuroscientists. The entire procedure takes about 30 minutes per rat.

Although most potential problems associated with general stereotaxic surgery have already been described [26,27], there are some critical points that need to be monitored for precise procedure execution. Proper placement of the rat in the stereotaxic device, along with accurate coordinates for targeting specific brain regions/structures is crucial for successful stereotaxic neurosurgery and obtaining high-quality electrophysiology results. To minimize brain tissue damage, it is important to slowly advance both stimulation and recording electrodes during their insertion into the targeted structures, particularly to avoid neuronal destruction. Throughout the surgery, it is essential to monitor specific animal signs such as breathing rhythm, withdrawal reflexes to tail and toe pinch, heart rate, and internal temperature control as described in previous studies [26,13].

Most potential problems with stereotaxic surgery have been described [26,27], but there are critical points to monitor for precise execution. Proper placement of the rat and accurate coordinates are crucial for successful surgery and high-quality results. Slowly advancing electrodes during insertion is important to minimize brain tissue damage. Monitoring animal signs such as breathing, reflexes, heart rate, and temperature control is essential [26,13].

Considering variations in strains, ages, adipose tissue content and inter-animal sensitivity to anaesthesia, animals may exhibit different sensitivities to the anaesthetic. The appropriate level of anesthesia should generally manifest within 1 to 5 minutes after injection. If not, one-tenth of the initial urethane dosage should be administered. The anaesthesia level should be maintained for the entire duration of the surgery, typically lasting 3-4 hours, although additional maintenance doses may be required in some cases. Therefore, it is highly recommended to plan pilot studies to determine the precise coordinates and correct dosage of the anaesthetic. Minimizing electrostatic noise interference is important and using a suitable faraday cage and creating an integrated grounded system is recommended.

The eLab electrophysiology system is versatile for both invasive and non-invasive, in-vivo, and in-vitro recordings of the central and peripheral nervous systems. This device utilizes a 24-bit analog-to-digital converter which allows for high-resolution signals with low gain amplification and reduced noise. Unlike other general equipment that may only perform specific techniques, the eLab can cover all extracellular techniques. This means that researchers can conduct a wide range of neuroscience and electrophysiology studies using just one device. The comprehensive software included with the eLab allows for precise adjustments such as high and low frequency filters. ePulse is a stimulus isolator used for deep brain stimulation and includes a professional mixer for designing stimulus patterns. Furthermore, eProbe is versatile software for monitoring and analyzing LFP signals, offering various analysis modes. eLab facilitates neuroscience and cardiovascular research employing various electrophysiological techniques. Many published papers have utilized eLab/ePulse devices for a wide range of electrophysiological research [9,10,[14], [28], [29], [30], [31]].

The neurosurgical stereotaxic process described in this study includes general steps and electrode insertion procedures. Also, protocol design and the order of running protocols for fEPSP recordings using ELab/ePulse can be applied to mice with some exceptions and modifications. Specific adaptors or other lab-made equipment are necessary to position mice on the stereotaxic device. It is important to note that the corneal blinking reflex may not be visible in rats. Due to anatomical differences, a separate brain atlas was created for mice, requiring precise measurement of target structure coordinates. Furthermore, there is no need for a correction coefficient. When conducting fEPSP recordings, adjustments to stimulation and recording parameters, such as pulse cycle and duration should be made.

This study outlines the steps of the neurosurgical stereotaxic process, including electrode insertion procedures. The protocol for fEPSP recordings using ELab/ePulse can be applied to mice with some modifications. Specific adaptors or lab-made equipment are needed to position mice on the stereotaxic device. The corneal blinking reflex may not be visible in rats. A separate brain atlas was created for mice due to anatomical differences, requiring precise measurement of target structure coordinates. No correction coefficient is needed. Adjustments to stimulation and recording parameters should be made when conducting fEPSP recordings.

## Conclusions

Performing *in vivo* extracellular local field potential recording involves inserting stimulatory and recording electrodes into specific structures. This requires precise stereotaxic neurosurgery to accurately place the electrodes and ensure high-quality EPSP recording. Conducting neurostereotaxic surgery with high accuracy not only yields superior results but also reduces the number of animals needed for the study. Moreover, using versatile and powerful neurophysiology experimental equipment like eLab/ePulse, ensures reliable results and experiment quality. By planning a systematic and precise stereotaxic neurosurgery procedure alongside robust and dependable neurophysiology experimental equipment, researchers can achieve guaranteed high-quality results.

## CRediT author statement

**Mansour Azimzadeh, King-Hwa Ling, Pike-See Cheah,** and **Parham Reisi:** conceived and designed the experiments; **Mansour Azimzadeh** and **Mohd Amirul Najwa Mohd Azmi:** performed the experiments; **Pike-See Cheah** and **Mansour Azimzadeh:** analyzed the data; **Mansour Azimzadeh** wrote the paper.

## Declaration of Competing Interest

The authors declare that they have no known competing financial interests or personal relationships that could have appeared to influence the work reported in this paper.

## Data Availability

Data will be made available on request.

## Appendix Supplementary materials

Image, application 1
