# Supplementary material for: Step-by-step approach: Stereotaxic surgery for in vivo extracellular field potential recording at the rat Schaffer collateral-CA1 synapse using the eLab system
Source: MethodsX. 2023 Dec 29;12:102544. doi: 10.1016/j.mex.2023.102544 (PMC10820282; doi:10.1016/j.mex.2023.102544)
Supplement: Supplementary file 1 [file mmc1.docx]

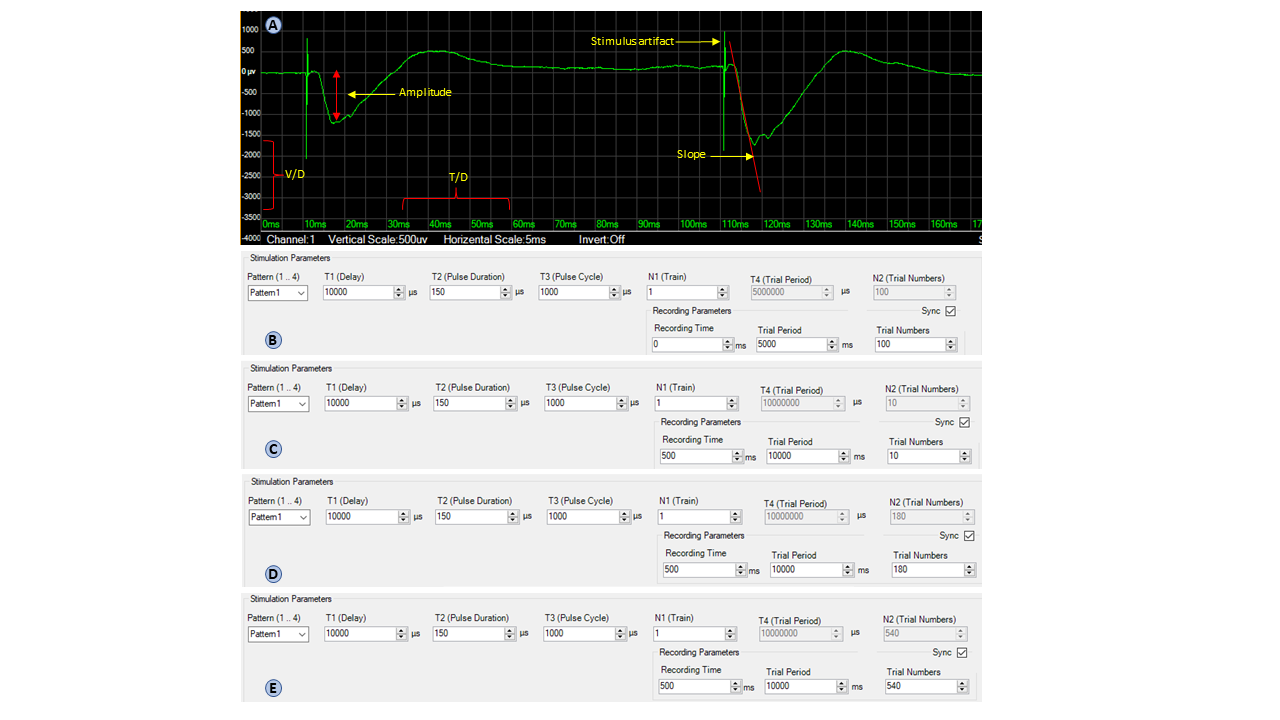


**Supplementary Figure 1. A)** This image depicts the eLab environment and defines the amplitude and slope of the EPSP. V/D stands for voltage division, while T/D stands for time division. The image also displays examples of designed protocols for **B)** Pattern test, **C)** IO function, **D)** Baseline 30 minutes, and **E)** Baseline 90 minutes.


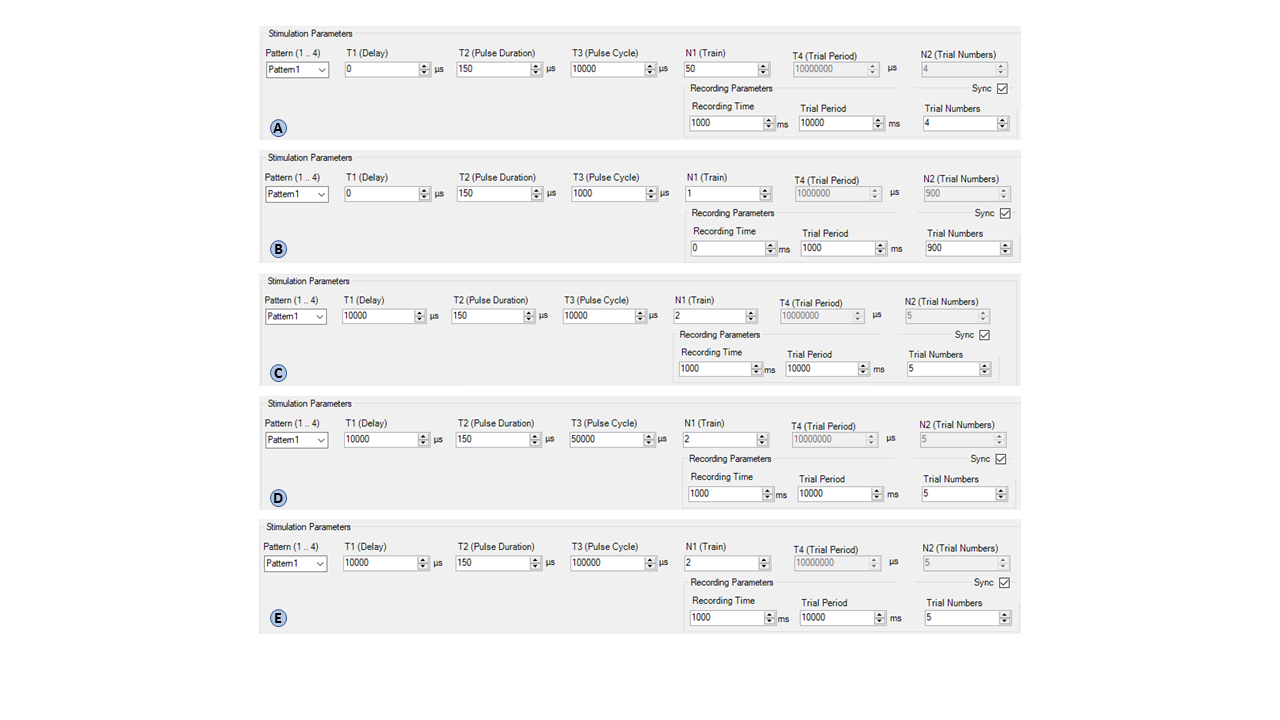


**Supplementary Figure 2.** Shows sample protocols for **A)** inducing LTP at 100 Hz, **B)** inducing LTD at 1 Hz, and **C-E)** paired pulses at 10, 50, and 100 ms respectively.
